# A Review on Traditional and Artificial Intelligence-Based Preservation Techniques for Oil Painting Artworks

**DOI:** 10.3390/gels10080517

**Published:** 2024-08-06

**Authors:** Salman Khalid, Muhammad Muzammil Azad, Heung Soo Kim, Yanggi Yoon, Hanhyoung Lee, Kwang-Soon Choi, Yoonmo Yang

**Affiliations:** 1 Department of Mechanical, Robotics and Energy Engineering, Dongguk University-Seoul, 30 Pil-dong 1 Gil, Jung-gu, Seoul 04620, Republic of Korea; salmankhalid@dgu.ac.kr (S.K.); muzammilazad@dgu.ac.kr (M.M.A.); 2 Korea Testing Certification, 22 Heungandaero-27-gil, Gunpo 15809, Gyeonggi-do, Republic of Korea; yanggi40@ktc.re.kr; 3 Con-Tech, School Based Enterprise, Industry-Academic Cooperation Foundation, Korea National University of Cultural Heritage, 367, Baekjemun-ro, Gyuam-myeon, Buyeo-gun 33115, Chungcheongnam-do, Republic of Korea; contech@nuch.ac.kr; 4 VR/AR Research Center, Korea Electronics Technology Institute, 11 World Cup buk-ro 54-gil, Mapo-gu, Seoul 03924, Republic of Korea; lenon@keti.re.kr (K.-S.C.); yym064@keti.re.kr (Y.Y.)

**Keywords:** oil paintings, maintenance practices, preservation techniques, traditional methods, artificial intelligence, gel-based cleaning

## Abstract

Oil paintings represent significant cultural heritage, as they embody human creativity and historical narratives. The preservation of these invaluable artifacts requires effective maintenance practices to ensure their longevity and integrity. Despite their inherent durability, oil paintings are susceptible to mechanical damage and chemical deterioration, necessitating rigorous conservation efforts. Traditional preservation techniques that have been developed over centuries involve surface treatment, structural stabilization, and gel-based cleaning to maintain both the integrity and aesthetic appeal of these artworks. Recent advances in artificial intelligence (AI)-powered predictive maintenance techniques offer innovative solutions to predict and prevent deterioration. By integrating image analysis and environmental monitoring, AI-based models provide valuable insights into painting preservation. This review comprehensively analyzes traditional and AI-based techniques for oil painting maintenance, highlighting the importance of adopting innovative approaches. By integrating traditional expertise with AI technology, conservators can enhance their capacity to maintain and preserve these cultural treasures for future generations.

## 1. Introduction

Oil paintings endure as timeless treasures, serving as windows to the past, expressions of cultural identity, and embodiments of artistic excellence [1]. Across centuries, they have borne witness to historical events, encapsulated the depths of human emotion, and immortalized the beauty of landscapes and portraits [2]. Beyond their aesthetic appeal, oil paintings have immense cultural and historical significance, providing tangible links to eras and civilizations that have long gone. From masterpieces of the Renaissance to modern works, these artworks offer invaluable insights into the evolution of artistic techniques, societal values, and cultural norms [3]. Preserving oil paintings goes beyond protecting physical objects; it represents a commitment to safeguard the rich tapestry of human creativity and cultural heritage for generations [4].

Oil painting is a form of painting that is created by the rapid drying of oil and canvas. Owing to the peculiarity of the oil-painting pigment, a good luster can be maintained for a long time [5]. Although oil paintings are revered for their durability and longevity, they are not immune to various forms of deterioration and damage [6]. Over time, these artworks may suffer from a multitude of failures that compromise their integrity and aesthetic appeal [7,8,9]. One common issue is the development of cracks and flaking caused by the contraction and expansion of the painting surface, owing to fluctuations in temperature and humidity [10]. Additionally, exposure to light can lead to the fading and discoloration of pigments, thereby altering the original appearance of the artwork [11,12]. Environmental pollutants, such as dust and dirt, can accumulate on painting surfaces, dulling their sheen and obscuring details. Furthermore, improper handling and storage practices can result in physical damage, such as tears or punctures in the canvas. Inadequate restoration efforts, or the use of inappropriate materials, may exacerbate existing issues or introduce new problems. Overall, to mitigate the risk of these failures and ensure the longevity of these valuable cultural artifacts, the preservation of oil paintings requires careful attention to various factors [13].

Preserving and conserving oil paintings is a meticulous and multifaceted process that draws on a rich tradition of practice and technique. Traditional methods of maintaining and conserving oil paintings have evolved over the centuries, guided by a deep understanding of materials, craftsmanship, and artistic intent [14,15]. Surface cleaning is a fundamental practice in the conservation of oil paintings. This process involves the careful removal of accumulated dirt, grime, and discolored varnish layers from the painting’s surface using gentle solvents and cleaning agents. Surface cleaning enhances the visual clarity and vibrancy of the artwork while also preventing further deterioration from environmental pollutants. Various surface cleaning methods have been used in literature; however, among these methods, the gel-based cleaning method is the most commonly employed for the preservation of oil paintings [16,17]. Another crucial aspect of oil painting conservation is the stabilization of the support structure, typically a canvas or wooden panel [18]. This may involve addressing issues such as tears, punctures, or deformations in the support, as well as reinforcing weak or fragile areas to prevent structural failure. Conservation specialists employ a range of techniques, including patching, lining, and structural repairs, to ensure the long-term stability of a painting’s support [19,20]. In addition to surface cleaning and structural stabilization, conservators often undertake retouching and inpainting to address areas of loss or damage in the paint layer. This delicate process requires a thorough understanding of the artist’s original techniques and materials, to ensure that the restoration work is in harmony with the rest of the painting. Conservators use carefully selected pigments and binding agents to recreate missing or damaged areas and seamlessly integrate them into the surrounding paint layer. Furthermore, preventive conservation measures play a crucial role in maintaining the condition of oil paintings over time. These may include controlling environmental factors, such as temperature, humidity, and light exposure, to minimize the risk of deterioration [10,21]. Appropriate handling, storage, and display practices also contribute to the long-term preservation of oil paintings, ensuring that they remain accessible to future generations. Overall, traditional practices in oil painting conservation embody a blend of scientific knowledge, artistic skills, and meticulous craftsmanship. By adhering to established conservation principles and techniques, conservators can safeguard the integrity and beauty of these precious cultural artifacts for years.

In recent years, the integration of artificial intelligence (AI) technology has revolutionized the field of oil painting maintenance, offering innovative solutions that complement and, in some cases, surpass traditional approaches [22]. AI-based methods utilize the power of machine learning algorithms and image analysis techniques to address various challenges in the preservation and conservation of oil paintings. A significant advantage of AI-based approaches is their ability to automate and streamline certain aspects of preservation processes. For example, AI algorithms can analyze high-resolution images of paintings to detect and classify different types of damage, such as cracks, tears, and discoloration, with remarkable accuracy and efficiency [23,24,25]. This automated analysis saves time and labor, while also allowing conservators to more effectively identify and prioritize areas in need of attention. Moreover, AI-based techniques offer a level of precision and consistency that is often difficult to achieve using traditional methods. By training machine learning models on large datasets of annotated paintings, AI systems can learn to recognize subtle patterns and features that are indicative of damage or deterioration. This capability enables AI algorithms to provide detailed and objective assessments of painting conditions, helping conservators to make informed decisions regarding appropriate treatment strategies. Another key benefit of AI-based approaches is their potential to enhance the reproducibility and standardization of conservation practices. Traditional conservation methods often rely heavily on the expertise and subjective judgment of individual conservators, which can lead to variability in the outcomes. In contrast, AI algorithms operate based on predefined rules and criteria, ensuring a more consistent and systematic approach to painting maintenance. Furthermore, AI technology offers novel capabilities for predictive maintenance and risk assessment in oil painting conservation. By analyzing historical data on painting conditions and environmental factors, AI models can forecast potential deterioration trends and identify areas that are at high risk of damage [26]. This proactive approach allows conservators to implement preventive measures, such as adjusting environmental conditions or scheduling maintenance interventions, to mitigate the risk of future deterioration. Overall, modern AI-based approaches represent a promising frontier in the field of oil painting maintenance, offering unprecedented capabilities for automated analysis, precision, reproducibility, and predictive maintenance [27]. While traditional methods continue to play a vital role in conservation practices, the integration of AI technology promises to enhance the efficiency, accuracy, and effectiveness of preserving these invaluable cultural artifacts for future generations.

**Figure 1:**
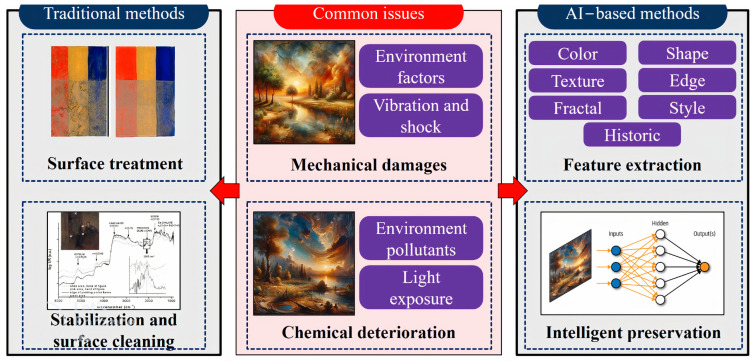
Overview of traditional and AI-based methods to address mechanical damage and chemical deterioration in oil paintings.

This study offers a comprehensive overview of preservation techniques for oil paintings, addressing common issues, such as mechanical damage and chemical deterioration. It covers key traditional methods that include surface treatment, structural stabilization through canvas reinforcement, and meticulous surface cleaning. Additionally, the study explores various AI-based approaches, emphasizing their capabilities in automated analysis, precise damage assessment, and intelligent preservation through predictive maintenance. Furthermore, the article delves into the specific imaging features crucial for training AI models effectively, as shown in Figure 1. The discussion concludes by highlighting future directions and challenges, underscoring the potential of AI technology and interdisciplinary collaboration to significantly advance the preservation of cultural heritage for future generations.

## 2. Common Issues in Oil Painting Preservation

Oil paintings are susceptible to various forms of damage that can be broadly categorized into two main types: mechanical damage and chemical deterioration. Mechanical damage refers to physical alterations caused by external forces, such as impacts, vibrations, changes in temperature, and fluctuations in humidity [21,28]. This damage may include cracks, tears, flaking, and the warping of canvas or support structures. However, chemical deterioration results from the gradual degradation of painting materials owing to chemical reactions with environmental pollutants, light exposure, and improper handling or storage conditions. This type of deterioration can lead to color change, fading, discoloration, and the breakdown of paint layers over time. Understanding and addressing both mechanical damage and chemical deterioration are essential for the preservation and conservation of oil paintings, ensuring their long-term stability and integrity.

### 2.1. Mechanical Damage in Oil Painting

This subsection thoroughly examines the impact of various mechanical factors, including environmental elements, such as relative humidity and temperature, as well as vibration and its impact on the integrity and preservation of oil paintings. Each factor is thoroughly explored to comprehend its contribution to mechanical damage and subsequent deterioration of these artworks.

#### 2.1.1. Environmental Factors (Relative Humidity and Temperature)

The impact of environmental factors, specifically relative humidity (RH) and temperature, on the deterioration of oil paintings is crucial for their preservation and conservation [29,30,31]. Fluctuations in RH can induce significant mechanical stress on various layers of a painting, including the paint and ground layers, as well as underlying supports, such as canvas or wood. High RH levels prompt hygroscopic materials to absorb moisture and expand, whereas low RH levels lead to contraction, potentially causing cracks, delamination, and warping. Therefore, maintaining a stable RH environment is essential for oil painting conservation, which requires a delicate balance between preservation requirements and energy consumption for climate control. Temperature also plays a pivotal role in the conservation and degradation of oil paintings. Unlike RH, temperature directly affects the chemical and physical properties of painting materials. Extremes in temperature accelerate chemical reactions within the paint layers, accelerating degradation and altering their appearance. High temperatures can increase the susceptibility to fading, yellowing, or brittleness, compromising integrity. Conversely, prolonged exposure to low temperatures may render materials brittle and prone to damage, such as cracking or delamination. Temperature fluctuations induce stress within the painting structure, potentially causing warping, distortion, or detachment of paint layers, particularly in rigid supports, such as wooden panels. Efforts to maintain a stable temperature environment are crucial for the conservation of oil painting. Museums and conservation facilities employ sophisticated climate control systems to regulate exhibition and storage temperatures, with the aim of minimizing variations and mitigating structural damage risks. By understanding and managing the combined effects of RH and temperature, conservators can ensure the long-term preservation of the aesthetic and historical value of oil paintings.

**Figure 2:**
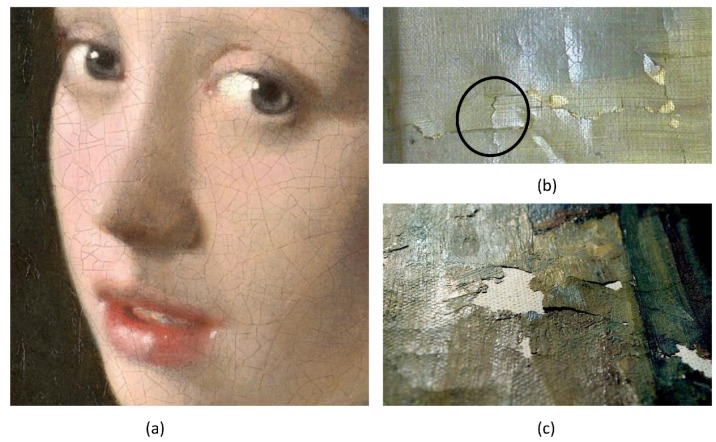
Common fracture phenomenon in historical paintings. (**a**) Girl with Pearl Earring. (**b**) Still life with flowers by Ambrosius Bosschaert. (**c**) Anonymous Italian [32].

Bosco et al. [32] investigated moisture-induced cracking in flexural bilayer systems, which is a phenomenon particularly relevant to historical paintings. The study examined how moisture content variations affect damage, focusing on crack channeling within brittle bilayers composed of a coating on a finite substrate. Three fracture mechanisms were identified: channeling cracks without delamination, channeling cracks with finite-length delamination, and channeling cracks with unbounded delamination. The study developed failure mechanism maps to analyze stress distribution, emphasizing factors like stiffness mismatch and interfacial toughness. By comparing simply supported and rigidly supported bilayers, the research highlighted the role of bending in crack propagation and suggested that delamination could be prevented by ensuring that the delamination toughness exceeds that of the paint layer. These observations provide insightful advice to conservators on how to control interior humidity and temperature to lessen the effects of crack channeling and delamination in historical paintings. Figure 2 depicts these fracture situations and also offers visual representations of common mechanisms seen in historical paintings: Johannes Vermeer’s “Girl with a Pearl Earring” (a) shows a network of channeling cracks on the paint surface; “Still life with flowers” (b) shows a crack that is kinked at the paint/substrate interface, resulting in two opposing delaminations; (c) shows a continuous delamination crack that eventually causes paint flaking.

Janas et al. [33] investigated the effects of temperature and relative humidity (RH) on the dimensional changes and mechanical properties of oil paintings. The study examined tensile characteristics, such as the modulus of elasticity and strain at break, focusing on the changing molecular composition of oil paint layers as they aged into solid films. The study discovered that oil paints exhibited increased elastic behavior and decreased plastic deformation over time, becoming stiffer and more brittle around 30 years after drying, using samples from Mecklenburg’s Paint Reference Collection. These results contradict existing physical models by implying that certain paints may become more brittle than ground layers made of glue, making them more prone to cracking. The study also assessed the shrinkage of oil paint resulting from organic medium evaporation and molecular displacement during the drying and aging process. It was determined that even in the absence of RH or temperature variations, cumulative shrinkage, particularly when constrained by a stable substrate, can surpass paint strain at break and result in fracturing. In parallel, Jonah et al. [34] examined the mechanical properties of modern painting materials, specifically artists’ acrylic and alkyd paints, across different temperature and relative humidity (RH) conditions. In ambient conditions, acrylic paints showed high flexibility and were able to sustain large deformations, whereas alkyd paints were stiffer and stronger but less capable of deformation. Acrylic paints exhibited increased stiffness and strength at lower RH compared to moderate RH, with reduced stretching ability. At temperatures below 15 °C, especially at −30 °C, acrylic paints rapidly became brittle. These findings highlight how modern painting materials behave mechanically under varying environmental conditions, providing insights into their vulnerability to damage. In a related study, Zhang et al. [35] developed numerical models to simulate interfacial and channeling cracks in oil paint layers due to cyclic variations in relative humidity (RH). Using data from Knole House in Kent, the study analyzed the impact of annual, biannual, and monthly RH cycles on cracking. Channeling cracks were observed to initiate slightly earlier than interfacial cracks, with predictions suggesting initiation in an uncontrolled environment at around 120 years [35]. High-amplitude annual RH cycles had a significant influence on crack initiation while eliminating this variation could extend crack initiation time to over 400 years, thereby enhancing the longevity of panel paintings. Furthermore, Richard et al. [36] studied the effect of silica gel on panel paintings in microclimate packages, focusing on controlling humidity and potential damage from temperature changes. The packages were tested both with and without silica gel, using various types of silica gels, and the paintings were monitored during transport. It was found that although well-designed packages might not need silica gel, adding a small amount can be helpful if the package has a higher air exchange rate without increasing the risk of damage to the paintings. 

**Figure 3:**
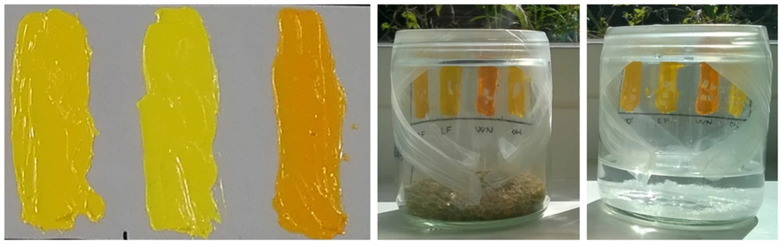
Curing of oil paint in varying RH conditions [37].

Modugno et al. [37] explored the impact of relative humidity (RH) on the chemical composition of modern oil paints during natural and artificial aging processes. The study involved analyzing oil paint layers that had aged naturally for a decade and subjecting them to additional aging under varying RH conditions, as shown in Figure 3. Significant changes in paint chemistry were observed, with high RH levels resulting in increased formation of dicarboxylic acids and higher hydrolysis compared to low RH. These insights into how RH influences the chemical evolution of oil paints are crucial for preservation and conservation practices. For further exploration, interested readers may refer to related studies [9,10,38]. 

#### 2.1.2. Vibration and Shock

**Figure 4:**
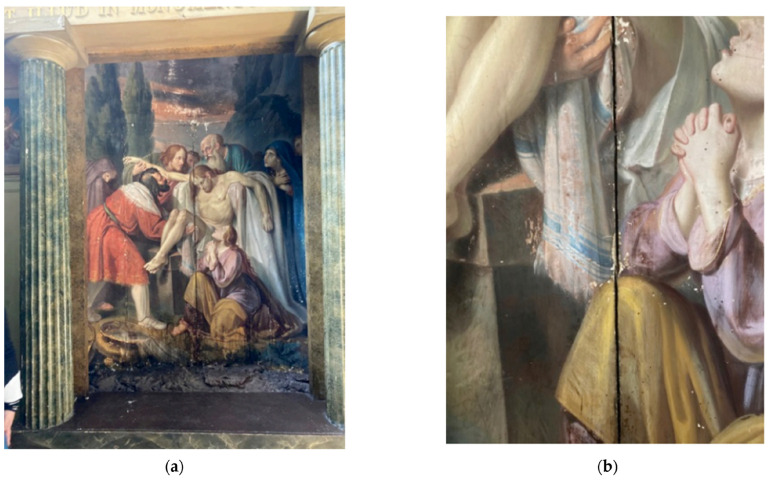
A (**a**) real painting and (**b**) a close-up, with a microclimate monitoring system attached inside the inlet [41].

Vibrations and shock are significant mechanical threats to the integrity of oil paintings. Vibrations, often caused by nearby construction, transportation, or even heavy foot traffic, can induce stress within paint and canvas [39,40]. These repeated small movements lead to microcracks in the paint layers and weakening of the adhesive bonds between the paint and the canvas or support structure, eventually resulting in flaking or detachment of the paint. Similarly, shocks, such as those from accidental knocks, falls, or improper handling, can cause immediate and severe damage. Such shocks can lead to tears, punctures, or significant deformation of the canvas and the underlying support. Furthermore, these forces can disrupt the layered structure of the painting, leading to the flaking or complete loss of paint in the affected areas. Understanding the effects of vibrations and shock is crucial to developing effective conservation strategies to preserve the structural integrity and visual appearance of oil paintings. Carlo et al. created an IoT measurement and monitoring system with an emphasis on cultural heritage conservation to evaluate the condition of artworks [41]. “The Deposition”, a Giuseppe Patricolo canvas painting from the 19th century that is housed in the Santa Caterina Monastery in Palermo, Italy, was monitored using their system. Stress and possible degeneration were monitored by measuring microclimatic elements like temperature, humidity, and vibrations. Figure 4 displays an up-close look at the integrated measurement system (Figure 4b), as well as the climatic monitoring device mounted on the painting (Figure 4a). To preserve the artifact, this method sought to include monitoring solutions within the work of art without causing any mechanical, chemical, or physical harm.

Yulong et al. [42] conducted an experimental study to analyze the vibration characteristics of canvas and primed canvas to prevent damage during transportation and slow their aging. Using an excitation mechanism and laser Doppler vibrometer, they investigated two dummy paintings in different orientations within a controlled climate box. The study focused on identifying modal parameters through experimental modal analysis, comparing them before and after applying primer. These findings validated a numerical model that accurately reconstructed eigenmodes, also exploring the impact of humidity and temperature on the eigenfrequencies of the paintings. Table 1 summarizes recent research on mechanical damage in oil paintings, offering insights and methodologies crucial for conservation practices.

### 2.2. Chemical Deterioration in Oil Paintings

Chemical deterioration of oil paintings results from complex interactions between the materials used in the artwork and various environmental factors. This type of damage is often gradual and can significantly alter the visual and structural integrity of paintings over time. The primary causes of chemical deterioration include environmental pollutants [43] and light exposure [44].

#### 2.2.1. Environmental Pollutants

Pollutants such as sulfur dioxide, nitrogen oxides, ozone, and particulate matter pose risks to artworks by reacting chemically with oil paint and varnish components, causing discoloration, loss of gloss, and increased brittleness in paint films. Moisture further facilitates the migration of harmful substances within paint layers. Abataleb et al. [45] investigated the effects of atmospheric pollutants on ancient paintings. The study measured pollutants such as RH%, temperature, and smoke and assessed their impact on colored materials made from earth pigments and linseed oil on canvas. Using techniques like EDX-SEM, XRD, FTIR, and spectrophotometry, the research found that pollutants led to discoloration and the development of a black dust layer and a calcified white mineral soap layer on the samples. These findings highlight the severe impact of environmental pollution on the preservation of cultural heritage. Laura et al. [46] examined the effects of sulfur dioxide (SO₂) and nitrogen oxides (NOₓ) on acrylic, alkyd, and styrene-acrylic paints under simulated environmental conditions. NOₓ caused significant damage, especially to acrylics, resulting in surfactant migration. Alkyd paints mostly suffered from hydrolysis, while styrene-acrylics showed varying degradation based on gas type and humidity. The analysis confirmed NOₓ’s strong oxidative effect and revealed different responses among paints and pigments, emphasizing the need for targeted preservation and more research on other pollutants. 

Anna et al. [43] examined the impact of air pollution on artworks at the Museum of Modern and Contemporary Art, analyzing soiling from four paintings to assess ionic content, lead, soot, and polynuclear aromatic hydrocarbons. Paintings stored since the mid-20th century showed elevated sulphate levels, possibly from indoor pollutants like plaster soiling and reactions with sulfur dioxide and calcite. In contrast, paintings stored more recently in urban areas exhibited higher calcium levels, likely influenced by local environmental conditions. Maria et al. [47] investigated microbial communities on a deteriorated oil painting, identifying both cultivable Firmicutes and non-cultivable Proteobacteria, emphasizing their roles in enzymatic degradation processes involving species like Penicillium and Eurotium. This underscores the microbial impact on artwork preservation and the need for targeted conservation strategies against biological threats. Similarly, Calderon et al. [48] conducted a multidisciplinary study on large-format Italian paintings at the National Theater of Costa Rica, assessing environmental conditions, quantifying fungal spores, identifying potential biodeterioration areas, and analyzing pigment distribution patterns. Their characterization of calcareous nannofossils in the ground layers of paintings provided insights into material age and preservation challenges in tropical climates. Terje et al. [49] modeled the effects of gaseous pollutants within microclimate frames (mc-frames) designed to protect artworks, showing higher concentrations inside mc-frames compared to outdoor environments. The study emphasizes the importance of reducing ventilation, minimizing mc-frame volumes, and incorporating pollutant absorbers to mitigate chemical degradation and preserve artworks effectively. 

**Figure 5:**
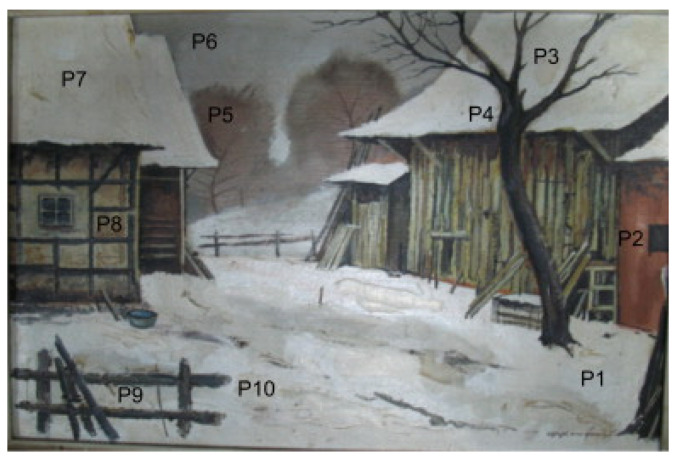
‘Winter’ painting by Gr. Alexandrescu used to demonstrate the extent to which gamma-ray treatment alters pigment colors [52].

Gamma radiation has been investigated for its potential in sterilizing oil paintings, particularly to address fungal contamination. Studies have shown that while gamma rays are effective in eliminating fungal spores, they generally do not cause significant color changes in the paintings [50]. In another study, this technique was applied to a 17th-century Peruvian painting that had been contaminated with mold and had previously undergone unsuccessful treatments. The study demonstrated that gamma radiation, when used at appropriate levels, successfully decontaminated the painting without damaging the original artwork or altering its pigments [51]. Maria et al. [52] evaluated gamma radiation treatment on the “Winter” oil painting by George Alexandrescu, using spectroscopic methods to analyze the painting before and after irradiation, as shown in Figure 5. The study found minimal changes in spectra and color, suggesting effective disinfection without significantly altering the painting’s chemical structure. 

#### 2.2.2. Light Exposure

Exposure to ultraviolet (UV) and visible light poses significant risks to oil paintings by initiating photochemical reactions that degrade binding media, leading to fading, yellowing, and increased brittleness. Organic pigments are particularly sensitive to light, undergoing noticeable color changes, while light exposure also triggers oil oxidation, further accelerating deterioration. Santiago et al. [44] exposed paints to seven different wavelengths in isolated aging cells equipped with LEDs and photodiodes to measure spectral reflectance. Temperature was regulated using thermocouples and fans to simulate exhibit conditions. They evaluated different exposure durations and levels of irradiation across ten series to develop a model correlating spectral radiant exposure with paint damage, aiming to quantify the impact of light exposure on these materials. 

Dang et al. [53] investigated the effects of prolonged exposure to museum lighting on the chromaticity of pigments commonly used in traditional Chinese heavy-color paintings. They tested three light sources: tungsten halogen with an infrared filter, metal halide, and an RYGB-type LED over 1152 h. The findings revealed that compared to the other sources, the RYGB-type LED induced the least average chromaticity shift, providing crucial insights into the design of museum lighting systems that preserve the colors of these delicate artworks. Ilaria et al. [4] studied the conservation issues of 20th and 21st-century oil paintings compared to older paintings. The main issues include fragile surface layers, paint sensitivity to water, solvents, and light exposure. These problems arise due to the chemical changes in the paint, like the formation of water-soluble salts and poor development of the oil binder. While gel-based cleaning can effectively remove surface dirt, it must be used carefully due to these sensitivities. The study emphasizes the importance of understanding the chemical makeup of modern oil paints to safely preserve them, especially when dealing with light exposure and cleaning.

Together, these studies underscore the importance of understanding light-induced deterioration mechanisms and implementing effective conservation strategies to safeguard artworks from irreversible damage caused by environmental exposure. Table 2 provides an overview of studies investigating chemical deterioration in oil paintings, summarizing methodologies employed, key findings, and implications for conservation practices.

## 3. Traditional Methods for Oil Painting Preservation

Given their susceptibility to mechanical damage and chemical deterioration, the preservation of oil paintings has been a focus for centuries. Traditional preservation methods have evolved alongside advances in conservation science and technology, often relying on techniques that mitigate environmental influences and physical threats to ensure the longevity and aesthetic quality of these artworks.

### 3.1. Surface Treatment Method

**Figure 6:**
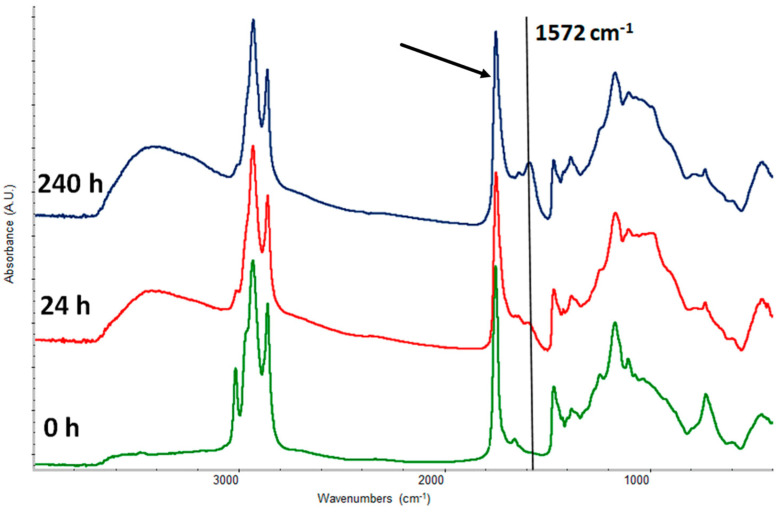
Representation of the kinetics of smalt carboxylate formation with a black arrow depicting the carboxylate band [58].

Surface treatment encompasses various methods that are crucial for oil painting preservation, with varnishing being the foremost technique. Varnishing is among the oldest and most effective methods for oil painting preservation [54,55]. Historically, natural resin varnishes, like dammar or mastic, were applied to provide a protective layer. Varnishes enhance color saturation, impart gloss, and shield the painting from environmental pollutants and UV radiation [56]. They also act as sacrificial layers that can be periodically removed and replaced. Modern synthetic varnishes offer enhanced UV protection and clarity, minimizing yellowing and discoloration over time. Osama et al. [57] investigated the use of zinc oxide (ZnO) nanoparticles as coatings for varnishing oil paintings on paper supports. The study aimed to assess how these nanoparticles protect against degradation. A coating containing 2% ZnO nanoparticles offered high transparency while significantly improving the durability of linseed oil-based paintings against UV aging, reducing color changes. Additionally, the coatings showed enhanced resistance to fungal attack by Trichoderma reesei and Aspergillus niger and reduced dirt accumulation after six months of outdoor exposure. This research highlights the potential of ZnO nanoparticles in varnishing to safeguard oil paintings from environmental and biological deterioration. Poli et al. [58] investigated the formation of metal soaps in oil paintings, focusing on carboxylic acids from drying oils reacting with pigment cations, such as smalt, lead white, and zinc white. The study examined how these reactions produce carboxylates that migrate and aggregate, altering the appearance and behavior of the paint film and complicating cleaning processes. Figure 6 represents the kinetics of smalt carboxylate formation with a black arrow representing the carboxylate band. The study specifically explored the reactivity of terpenic acids found in historical varnishes, like colophony, dammar, and mastic. The experiments were conducted to understand the kinetics of carboxylate formation using palmitic and abietic acids, demonstrating their interaction with potassium salts. This research highlights the significant role of terpenic acids in the formation of metal soaps and underscores the implications of varnish composition on paint preservation and restoration practices. 

### 3.2. Structural Stabilization

In structural stabilization methods, canvas is a primary component crucial for preserving oil paintings, playing a critical role in both structural integrity and aesthetic presentation [59]. Traditionally crafted from linen or cotton, canvas provides a resilient and flexible support for paint layers, absorbing stresses and allowing controlled expansion and contraction in response to fluctuations in temperature and humidity [31]. This flexibility is essential to prevent cracking and ensure the long-term stability of the artwork. The quality and preparation of canvas are pivotal considerations in conservation practices. High-quality canvas, properly prepared with priming layers, such as gesso, creates a stable and uniform surface that supports paint adhesion and longevity.

**Figure 7:**
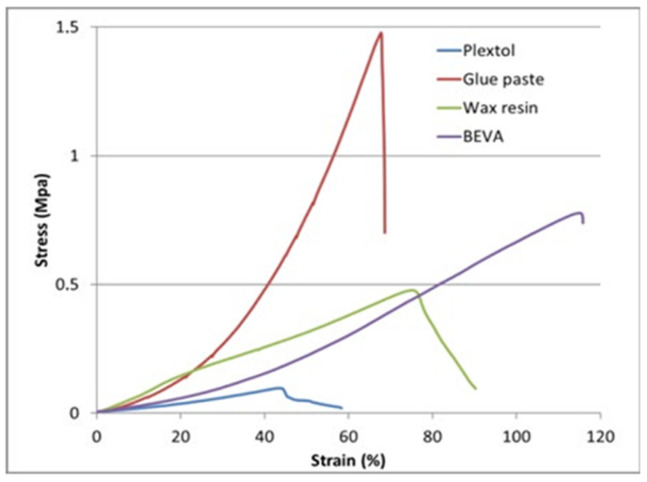
Comparison of the stress–strain curves of four adhesives [61].

Conservation efforts often entail meticulous cleaning and repair of the canvas to address dirt accumulation, stains, or tears that could compromise its structural integrity and the artwork’s visual coherence. Beyond its functional role, canvas texture and weave significantly influence the visual appearance of oil paintings. The texture can impact how light interacts with the paint layers, affecting color saturation and the overall aesthetic impression of the artwork. Conservation approaches frequently involve methods to stabilize and reinforce the canvas, such as lining with a secondary support fabric or applying consolidants to strengthen weakened areas without altering the original appearance [19]. Cecilia et al. [60] investigated the effectiveness of wax resin impregnation and lining as preservation methods for canvas paintings. The goal of the investigation was to determine how various treatments affected the diffusion of moisture into the canvas and the ensuing build-up of tension. The study revealed that while wax resin impregnation slowed down moisture uptake compared to untreated linen, tension from moisture-induced swelling occurred at different relative humidity levels. Factors such as canvas weave and thread density were identified as crucial in determining the susceptibility of paintings to climate-induced shrinkage damage. Additionally, the research found that Berger’s Ethyl Vinyl Acetate (BEVA^®^371), applied as a gel, had a limited impact on reducing moisture absorption. BEVA^®^371 gel’s inability to effectively penetrate the canvas fibers resulted in a minimal reduction in moisture uptake. Poulis et al. [61] conducted an in-depth exploration into the thermal and mechanical properties of adhesives that are crucial for the preservation of canvas paintings, particularly focusing on the lining process. The study delves into the comparative analysis of four commonly employed adhesives, including traditional formulations like animal glue–wheat flour paste (GP), wax resin, and innovative synthetic options, such as BEVA and Plextol polymers, as shown in Figure 7. By scrutinizing parameters such as glass transition temperatures, melting points, static lap shear strength, and creep resistance, the research aims to provide conservationists with vital insights into selecting adhesives that can effectively reinforce the structural integrity of canvas artworks. This investigation underscores the importance of informed adhesive choices to mitigate environmental impacts and enhance the long-term preservation of canvas paintings.

### 3.3. Surface Cleaning Method

Surface cleaning is a foundational practice in the preservation of oil paintings, crucial for maintaining their appearance and safeguarding them against deterioration over time [16]. Conservators meticulously assess the painting’s surface to identify accumulated dirt, pollutants, and degraded varnish layers that obscure the artwork’s original colors and details [62]. Using a combination of dry-cleaning methods to remove loose dust and gentle solvent applications for more stubborn contaminants, conservators aim to reveal the painting’s true appearance without compromising its delicate paint layers. Each cleaning step follows stringent conservation ethics to ensure minimal intervention and maximum preservation of the painting’s historical and artistic significance. Continuous monitoring and evaluation throughout the cleaning process help conservators achieve optimal outcomes while safeguarding the painting’s stability, ensuring it remains a lasting testament to artistic heritage [63]. In general, two types of cleaning methods are used to preserve oil paintings. These include gel- and liquid-based cleaning methods. Gel-based methods involve the application of specially formulated gels that adhere to contaminants on the painting’s surface, offering a controlled and gentle cleaning approach. On the other hand, liquid-based methods utilize solutions, which are applied directly to the painting to dissolve and remove specific types of dirt, varnish, or other surface deposits.

#### 3.3.1. Gel-Based Cleaning Methods

Lena et al. [64] examined cutting-edge techniques for removing ingrained surface soiling from fragile historical items, with an emphasis on Edvard Munch’s Aula paintings. Three unique cleaning solutions were investigated in the study: hydrogels from the Peggy series and Nanorestore Gel^®^ Dry, soft particle blasting, and CO_2_-snow blasting [65,66,67]. The conditions of the artworks were replicated using accelerated-aged mock-ups of unvarnished oil paint and chalk–glue grounds. When compared to dry polyurethane sponges used in prior cleaning campaigns, the Nanorestore Gel^®^ series exhibited promising efficacy in soiling removal and reduced pigment loss on water-sensitive surfaces. However, visual and analytical assessments conducted before and after cleaning produced variable outcomes. Carretti et al. [68] proposed a new class of organogels called ammonium carbamate form (PEICO_2_). The utility of PEICO_2_ as the gel is examined by cleaning historic oil-painted artworks. The developed gel combined features of both liquids and traditional gels, effectively removing surface patinas like dammar and acrylic polymeric resin from artworks without damaging the underlying paint layers. Analytical tests, including contact angle, FTIR measurements, and visual comparisons, confirm their efficacy in restoration, offering a versatile and efficient method for conserving artworks with aged surface coatings. Poly vinyl alcohol (PVA) has been used to develop novel twin-chain polymer network (TC-PNs)-based gels with excellent porosity and adhesion properties [69]. These gels are capable of accommodating aqueous solutions and oil-in-water microemulsions, further expanding their versatility in conservation practices. This capability makes TC-PNs particularly suited for cleaning modern and contemporary masterpieces by artists like Jackson Pollock, Pablo Picasso, and Roy Lichtenstein, ensuring preservation without damage by removal of soil from challenging, textured painted surfaces where traditional rigid gels may struggle. Porpora et al. [70] synthesized polydimethylsiloxane (PDMS) organogel sponges using sugar leaching techniques to vary pore size and distribution, influencing their solvent uptake and retention capabilities. Compared to non-porous PDMS slabs, sponge structures templated with sugar cubes exhibited higher porosity (77%) and larger pores (ca. 300 μm), whereas those templated with powdered sugar showed lower porosity (ca. 10%) and smaller pores (ca. 75 μm). These porous organic polymers (POPs) demonstrated versatile solvent absorption across a range of polarities, while rheological analysis indicated minimal impact on their gel-like properties. Application tests on mock-ups of painted artworks highlighted PDMS sponges as promising tools for controlled and selective cleaning artworks for their conservation.

#### 3.3.2. Liquid-Based Cleaning Methods

**Figure 8:**
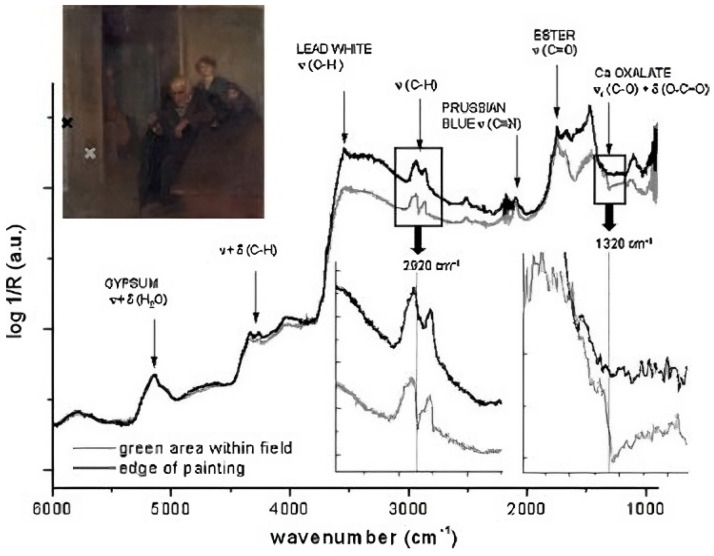
Areas of the painting analyzed using mid-FTIR spectroscopy showing the absence of Prussian blue in varnished regions (head, hand) and its presence under the frame where varnish is absent, confirming the original pigment use to depict a darkened interior [71].

Working closely with a painting conservator, Kenza et al. [71] used mid-FTIR fiber-optic reflectance spectroscopy to track an oil painting’s cleaning procedure using triammonium citrate solution. The elimination of a terpenic varnish component and calcium oxalate from the painting’s surface was verified by on-site results. Figure 8 shows certain portions of the artwork where Prussian blue is not visible. Prussian blue was specifically absent from the spectra obtained from varnished regions, like the man’s hand and head, while it was visible beneath the frame in areas without varnish. The discovery suggested that Prussian blue was first included in the painter’s palette for the application of the paint layers, probably to improve the image of the interior, which was shown as being dark and gloomy. These results were corroborated by laboratory FTIR and GC–MS analysis of cotton swabs used for cleaning, which provided information on the mechanism and effectiveness of the cleaning agent in the environment. Similar to this, Nikita et al. [72] investigated cleaning a Dutch oil painting from the eighteenth century called “Vision of Saint Lutgard of Tongeren” after removing the varnish and observing a gray, lead-rich salt crust. By utilizing elemental analysis in conjunction with scanning electron microscopy, they were able to verify that the crust was a separate layer that overlaid the paint, influencing its saturation, color, and appearance. The paper explores theories on the crust’s formation through material characterization, past treatments, and environmental factors. It details tests with various cleaning agents and techniques, highlighting Ethylenediaminetetraacetic acid (EDTA) solution as the most effective in removing the crust, with pH adjustments tailored to the paint color. Visual and analytical assessments post-cleaning demonstrated significant improvement in the painting’s appearance, validating the efficacy of the treatment. Table 3 provides a structured overview of various traditional methods employed in oil painting preservation, including the studies focusing on each method, key findings, and implications for conservation practices. It aims to assist conservators and museum professionals in making informed decisions to safeguard oil paintings from degradation, and ensure their long-term preservation.

## 4. AI-Based Preservation of Oil Paintings

This section introduces the use of AI in art preservation, discusses how AI utilizes oil painting features for intelligent vision, and explores case studies and examples that illustrate the effectiveness of AI in predicting artwork conditions. 

### 4.1. Introduction to AI in Art Preservation

Due to changing ambient environment conditions and forgery issues, it is challenging to always use traditional methods for the maintenance of oil paintings. This is because the changing ambient environment conditions can lead to humidity, pigment color degradation, paint loss, stains, and even cracks, which can distort the color performance and integrity of paintings [73,74]. Meanwhile, the oil painting may be further damaged by contaminants in the form of chemicals in the ambient, which can have a damaging effect on the color and detail of the painting. Therefore, it is imperative to investigate a scientific and efficient approach and develop suitable maintenance strategies without relying entirely on conventional subjective methods. AI technology enables precise analysis and monitoring of the condition of the paintings, providing data-driven understandings that enhance preservation techniques and ensure the authenticity and longevity of painted artworks [75,76]. The intelligent preservation of oil painting is accomplished by integrating scientific features and literature, color recognition, and intelligent inspection to identify any damage or cracks [77,78].

**Figure 9:**
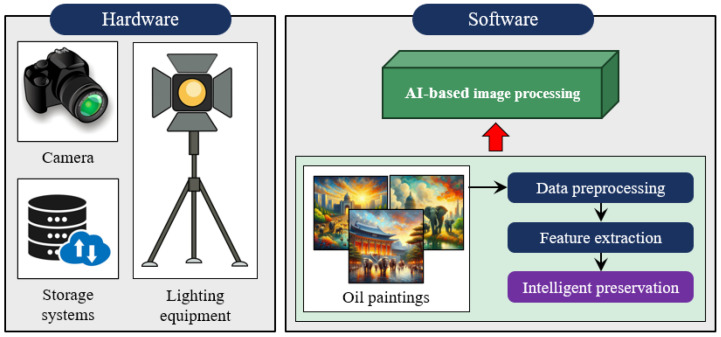
Some basic hardware and software components required for the AI-based maintenance of oil paintings.

Vision is the primary means by which oil painting elements and contents are perceived. Recently, vision perception has been enhanced through the integration of information technology in numerous computer vision [79,80] and damage identification tasks [81,82]. An analogous concept is implemented to enhance the speed and efficacy of oil paintings through an intelligent recognition system by substituting for human eye recognition. Figure 9 illustrates the hardware and software components of this recognition system, which focuses on intelligent vision-based preservation technology for oil paintings. The hardware is mainly comprised of a high-resolution camera, lighting equipment, and storage systems with capabilities essential for the detailed capture of painting features. The software system contains AI-based image-processing algorithms that can process and analyze the paintings. Through the integration of hardware and software, the main aim of intelligent preservation is to predict the deterioration pattern in the painting, authenticate the artwork, enhance artwork for failure recognition, and classify the artistic styles. The following sub-sections present details of these techniques and their role in the visual detection of oil paintings using AI.

#### 4.1.1. Predicting Deterioration Patterns

Oil paintings change in appearance from the time they are created in the artist’s studio. The effect is a consequence of the drying and natural aging processes that commence immediately after the application of the paint and gradually advance over time, excluding any alterations due to human actions [83]. The aging and degeneration of painted works of art involve alterations in the chemical, physical, and optical characteristics of the paint. Colors may experience fading or complete disappearance, paint may become increasingly translucent and yellow, resulting in a darker appearance, and thin layers may form on the paint surface, leading to various manifestations. Studies have also shown the temperature-related degradation of the vivianite pigment generally used for oil painting [84]. Similarly, the oil paintings have also been monitored for ambient conditions, and fungal aerial spores are determined to identify the areas of their possible biodeterioration [48]. All these deterioration patterns are crucial to understanding the degradation of oil paintings. However, manually monitoring these patterns is a tedious task requiring much effort and resources. Therefore, the utilization of AI can provide proactive means to preserve the characteristics of oil paintings, thereby extending life and maintaining the integrity of valuable artworks.

#### 4.1.2. Authenticating Artworks

**Figure 10:**
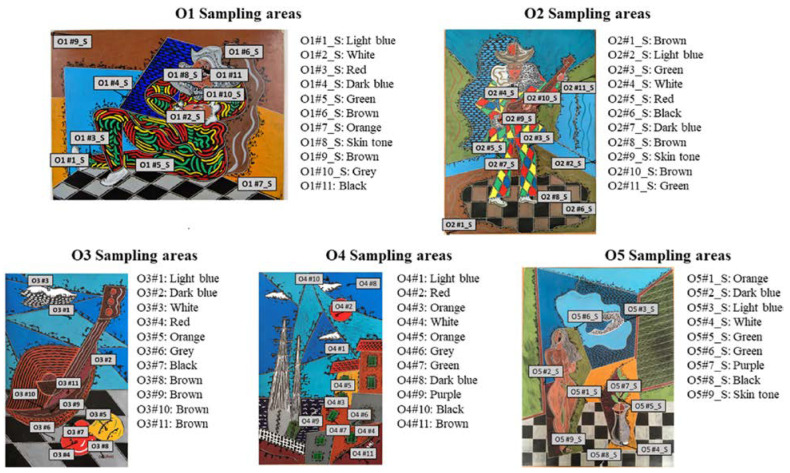
The five examined paintings fabricated by Oriani and the locations of the sites that were analyzed using XRF, μ–Raman, and FTIR spectroscopy (taken with permission from Ref. [86]).

In recent times, effort has been made to detect fraud in oil paintings by utilizing advanced techniques in computer vision and image processing. While intelligent techniques such as deep learning and machine learning have shown promising outcomes, further study is needed to develop an AI model that is specifically designed for detecting forgeries. One option that can potentially authenticate artworks is the use of image-to-image translation technologies, such as conditional generative adversarial networks (CGANs), to identify the unique brushstroke style of painters [85]. In the past, Manfrini et al. created the initial scientific database to assist in the verification of painting artworks [86]. Utilizing a combination of imaging and spectroscopic techniques, this database has identified a diverse array of organic dyes and inorganic pigments, with binders such as wax and oil verifying the utilization of the encaustic process on cardboard. Non-destructive testing (NDT) techniques, such as X-ray fluorescence spectroscopy (XRF), ultraviolet imaging, infrared (IR) and IR-false color photography, μ–Raman, and Fourier transform infrared spectroscopy (FTIR), have been used to identify fake paintings, as shown in Figure 10. Additionally, X-ray imaging techniques, such as laminography [87] and digital radiography [88], provide detailed insights into the underlying layers and structural composition of artworks, helping to reveal hidden alterations or forgeries. Furthermore, multispectral imaging [89], which covers a range from infrared to ultraviolet (UV) wavelengths, allows for a comprehensive analysis of the materials and techniques used in the paintings, offering critical data for authentication and conservation purposes. Although the comprehensive identification of all the materials utilized in the fabrication process of Oriani’s counterfeit paintings is advantageous, the extension of such approaches to other paintings requires excessive experimental resources and experience. Thus, it is important to identify potential AI-based techniques that are specially designed to overcome the challenges related to forgery and the authenticity of oil paintings.

#### 4.1.3. Image Enhancement and Failure Recognition

**Figure 11:**
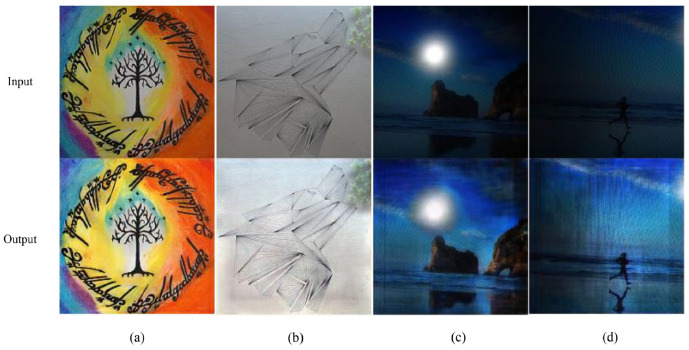
Results obtained from the proposed deep learning model for illumination correction using (**a**) oil pastel painting, (**b**) wall painting with vector information, and (**c**,**d**) images from a laptop screen (taken with permission from Ref. [90]).

The image enhancement process involves techniques that improve the visual quality of images, aiding in the detailed analysis, maintenance, and preservation of oil paintings. Enhanced images allow conservators to detect fine details, such as cracks, fading, or areas where paint may be lifting, which are not easily visible to the naked eye, thereby helping in early failure recognition of potential issues and enabling timely interventions to prevent further deterioration. Goswami and Singh [90] suggested a CNN-based deep-learning method to correct image illumination for paintings. The CNN model is validated on numerous images, such as oil pastel paintings, hand-drawn pictures, and images of a laptop screen, as shown in Figure 11. Chang [91] also employed a similar methodology that relies on the super resolution convolutional neural network (SRCNN) model and is based on a UNet-based architecture. The proposed model provided improved image resolution of oil paintings, overcoming the limitations of traditional approaches that disregard the distribution of image edges, which causes blurry outlines in the final images. These studies suggest the use of AI-based models to enhance the image resolution of oil paintings, consequently providing high-quality data for better failure recognition.

#### 4.1.4. Classifying Artistic Styles

**Figure 12:**
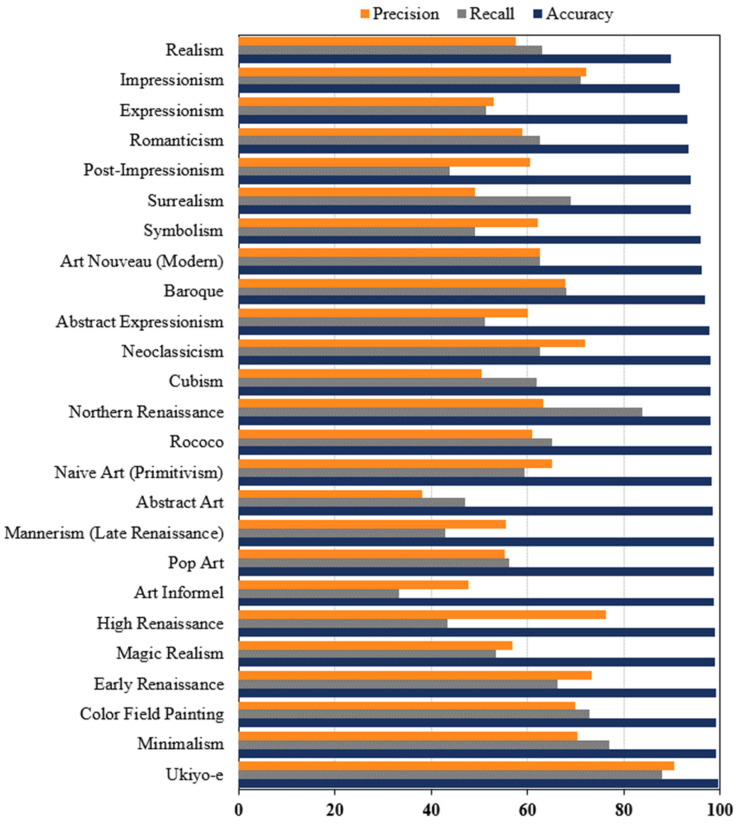
The accuracy, precision, and recall score for each art style in the dataset.

Extracting accurate classification features from oil paintings is difficult due to variations in painting techniques and content. However, if the artistic styles of various oil paintings could be captured efficiently, it could be beneficial for their preservation. The stroke feature, described as the distinctive marks or brushstrokes made by the artist, can significantly contribute to classifying various artistic styles. Therefore, the paintings can be classified utilizing edge detection to extract stroke features through machine learning models [92]. Similarly, Lecoutre et al. [93] classified various artistic styles of paintings using deep learning by first pre-training the model on ImageNet data and then fine-tuning the model to obtain artistic styles. The Wikipaintings dataset [94] used for this purpose comprises 25 different artistic styles containing both visual and historical information about the paintings. Figure 12 shows the accuracy, precision, and recall scores for each art style using the proposed approach. This signifies the prospect of using the pre-trained transfer learning models, especially when the failure modes are excessive and the available data for each failure type is limited. Pre-trained models of different artistic styles can be utilized to identify potential deterioration patterns in oil paintings. The damage in such cases can be identified through the change in edge features, style, and texture of the paintings.

### 4.2. Oil Painting Features for Intelligent Vision

**Figure 13:**
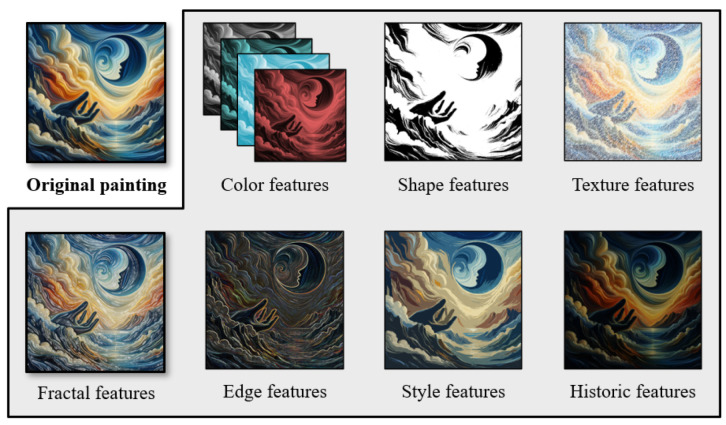
An original painting and some important features present in oil paintings that can be used for intelligent vision-based maintenance.

The implementation of intelligent vision for the preservation of oil paintings requires accurate detection and quantification of the various features that are shown in Figure 13. These features facilitate the understanding of intricate details, helping in conservation efforts and providing insights into the artist’s methods and materials. The next sub-sections highlight the salient features of oil paintings that are important in the AI-assisted maintenance of oil paintings to process the information accurately.

#### 4.2.1. Color Feature

Color features in oil paintings convey the artist’s expression, mood, and narrative, playing a vital role in the overall aesthetic impact of the painting [95]. Analyzing color composition aids in intelligent vision for the preservation of valuable artworks. In general, AI-based image recognition is performed using RGB color space; however, in the case of oil painting, HSV color space is used due to its advantage of judging the relationship between multiple colors [96]. The HSV color space primarily contains three components: hue (*H*), saturation (*S*), and value (*V*), which are built on the human perception of colors [97]. Therefore, the RGB color space in the oil painting is mostly transformed into the HSV color space [98], and the detailed conversion process is as follows:

(1) $$V=\;maximum\left(R,G,B\right),$$

(2) $$S=\;\frac{V-minimum(R,G,B)}{V},$$

(3) $$H=\;\left\{\begin{array}{c} \;5+B^{\prime },\;\;R=\mathrm{maximum}\left(R,G,B\right)\;and\;G=\mathrm{maximum}\left(R,G,B\right)\\ \;1-G^{\prime },\;\;R=\mathrm{maximum}\left(R,G,B\right)\;and\;G=\mathrm{maximum}\left(R,G,B\right)\;\\ \;1+R^{\prime },\;\;G=\mathrm{maximum}\left(R,G,B\right)\;and\;B=\mathrm{maximum}\left(R,G,B\right)\\ \;3-B^{\prime },\;\;R=\mathrm{maximum}\left(R,G,B\right)\;and\;B=\mathrm{maximum}\left(R,G,B\right)\\ \;3+G^{\prime },\;\;B=\mathrm{maximum}\left(R,G,B\right)\;and\;R=\mathrm{maximum}\left(R,G,B\right)\\ \;5-R^{\prime },\;\;R=\mathrm{maximum}\left(R,G,B\right)\;and\;G=\mathrm{maximum}\left(R,G,B\right) \end{array}\right\}.$$

For oil painting, *H*, *S*, and *V* are divided into eight, two, and one, respectively, as follows:

(4) $$H=\;\left\{\begin{array}{c} \begin{array}{c} \;0,\;\;H\epsilon (344,\;24)\\ 1,\;\;H\epsilon (24,\;54)\\ \;2,\;\;H\epsilon (54,\;107)\\ \;3,\;\;H\epsilon (107,\;164)\\ \;4,\;\;H\epsilon (164,\;219)\\ \;5,\;\;H\epsilon (219,\;274) \end{array}\\ \;6,\;\;H\epsilon (274,\;315)\;\\ \;7,\;\;H\epsilon (315,\;344) \end{array}\right\},$$

(5) $$S=\;\left\{\begin{array}{c} \;0,\;\;S\epsilon (0.20,\;0.66)\\ \;1,\;\;S\epsilon (0.66,\;1.00) \end{array}\right\},$$

(6) $$V=\;0,\;V\epsilon \left(0.14,\;1.00\right).$$

In the context of intelligent vision, the oil painting color is directly correlated with the wavelength of light. The variation in frequency and wavelength among several types of light allows for the expansion of the color range in oil paintings and enables the quantification of non-uniform intervals based on this distinction. In the above formula, the numbers 0 to 7 represent distinct tone categories [99]. During the extraction of color features, varying weights are assigned to the 3D feature vector of HSV to create a 1D feature vector, which facilitates further assessment. This 1D color feature vector contains color information of the paintings, which can be utilized for the AI-based preservation of oil paintings.

#### 4.2.2. Shape Feature

Shape features describe the geometric properties of objects within an image. These features emphasize the outline and form of the objects, such as boundaries, contours, and overall form, typically where the form of the object is crucial, such as object recognition and classification [95,100]. The fundamental characteristic of oil painting is shape, which is primarily identified and analyzed using form classification and contour feature retrieval [101]. The form and outline characteristics of oil painting are mostly expressed by line segments, while the local characteristics are displayed through color areas [102]. When painters create their artwork, they are impacted by their objective experiences. As a result, they develop distinctive characteristics in terms of both the area and perimeter, whose characteristics are derived from fundamental shape elements [103]. These features are considered to be more advanced than texture features due to their possession of specific qualities, such as uniqueness and geometric invariance. Furthermore, the area and perimeter aspects might somewhat reflect the qualities of the primary subjects depicted in paintings. Therefore, the shape features are necessary to perform the AI-based prevention of damage to oil paintings and can provide rich information regarding the existing damage present in oil paintings. Any distortion in oil paintings can be identified using the shape features, and advanced AI methods can also quantify the damage severity using shape features and their derivatives.

#### 4.2.3. Texture Features

**Figure 14:**
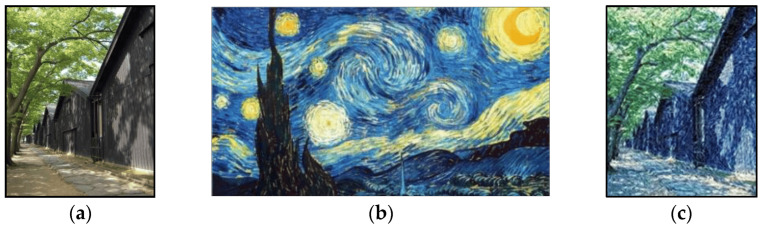
(**a**) Landscape, (**b**) oil painting of Van Gogh’s “Starry Night”, and (**c**) stylized image (taken with permission from Ref. [106]).

Texture features describe the surface properties and patterns in the oil paintings. These features primarily address the visual patterns, granularity, and structural arrangement of the surface, which can be repetitive or random. Texture features are useful to distinguish between regions of the paintings based on their surface appearance, such as identifying materials or surface conditions. Methods to extract texture features include statistical measures (like dissimilarity, correlation, and entropy), filter-based approaches (such as Gabor filters), and model-based methods (like Markov random fields) [104]. Texture metrics are valuable for image segmentation, feature extraction, and image classification [105]. Therefore, they have been used in impressionism-focused image stylization, which blends a source image with an oil painting texture of impressionism style to produce an impressionism-focused image, as shown in Figure 14 [106]. Numerous texture-based AI models have also been developed in the fields that specifically utilize the CNN models [107]. This signifies the potential of textural features in oil paintings and how they can be utilized in the maintenance of oil paintings. These textural features can provide surface inspection of the oil paintings and help identify areas of the painting that have undergone changes or damage over time. 

#### 4.2.4. Edge Features

Edge features can provide detailed structural information about oil paintings that can help identify cracks, flaking, and other forms of physical damage in oil paintings. Edge detection in image processing identifies positions where there is a significant change in color value, delineating the dividing lines between different areas with distinct local characteristics [103]. By highlighting the boundaries and edges within the image, conservators can pinpoint areas that need restoration. Edge detection is found crucial in achieving accurate and content-aware style transfer for oil paintings. It helps preserve the semantic information of the image while effectively imitating the intended artistic style [91]. Image sharpening is accomplished by employing the Laplace operator, which improves the distribution of edges and resolves the problem of blurred contours commonly encountered in traditional techniques. Therefore, edge features are one of the important features for intelligent vision that is capable of providing specific information from oil paintings that can be used to quantify the authenticity of oil paintings [101], as well as identifying craquelures in old oil paintings [108]. 

#### 4.2.5. Fractal Features

**Figure 15:**
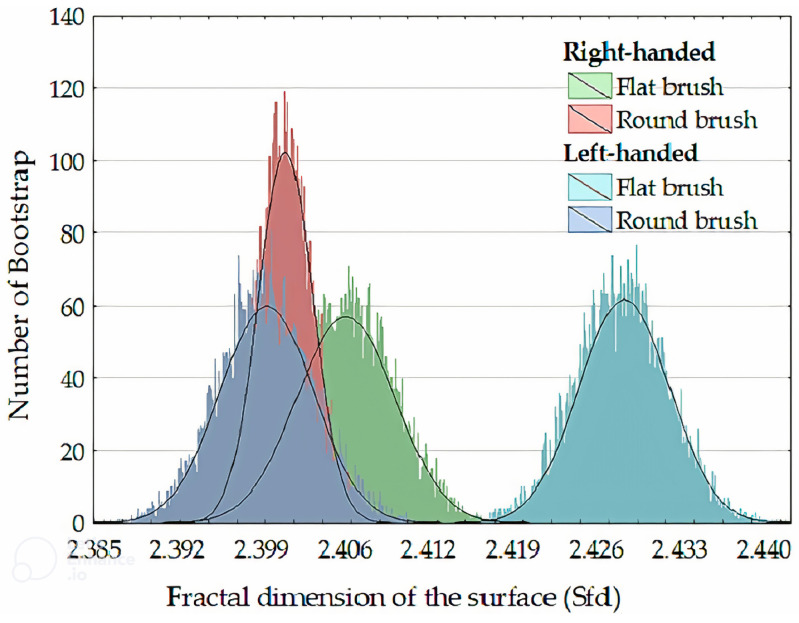
The fractal dimension distribution based on right- and left-handed painters using a flat or round brush replicated by Bootstrap [111].

Fractal geometry is a mathematical discipline that focuses on analyzing intricate patterns seen in irregular geometric shapes. Fractals are geometric objects that exhibit self-similarity at various scales [109]. Fractals can be employed to characterize image textures when the spatial distribution of local image textures displays irregular shapes. The fractal geometry is often measured using a fractal dimension that differs from the dimension defined in Euclidean space [105]. Bigerelle et al. used fractal dimensions to characterize brushstrokes on paintings [110]. The researchers determined that the brushstrokes on the paintings exhibit a fractal pattern, which may be described by the topographic slope parameter that distinguishes the various morphological structures of a painting. The fractal features of surface textures at different scales of individual brushstrokes, such as brush strokes, canvas filling, and canvas geometry, are important factors, as they reveal that painters’ styles encompass multiple scales of detail. Nevertheless, it is important to employ a filtering process to separate and isolate the distinct elements that are inherent in various physical occurrences. The fractal dimension has also been used to identify flat brush, round brush, right-handed brush stroke, and left-handed brush stroke, as shown in Figure 15 [111]. Thus, the fractal features can provide valuable insights for the AI-based maintenance of oil paintings. By characterizing the intricate patterns of brushstrokes at various scales and evaluating the change in fractal dimensions due to damage, AI algorithms can distinguish tones in the painting styles and aid conservators in effectively identifying and preserving these artworks.

#### 4.2.6. Style Features

The style features of the oil painting are particularly focused on either emotional expression [112] or the schools of artworks, such as Cubism, Baroque, and Impressionism [113,114]. Every painter strives to convey their unique emotional expression, allowing their distinctive style and cultural diversity to shine through in their artwork. As a result, it has become both the driving force behind the creation of paintings and a means of expressing the author’s inner spiritual world. Undoubtedly, creators residing in different environments possess distinct perceptions. Consequently, the techniques, subject matter, ideas, and modes of representation also vary significantly, and each artwork has developed its style by blending its characteristics. AI models can play a vital role in identifying these style features for the maintenance of oil paintings. Through the utilization of neural networks, the analysis of paintings can be extended, and various styles can be captured [115]. Each layer in a neural network acts as a nonlinear filter bank, with deeper layers capturing more complex style features. Therefore, DenseNet–121, ResNet–50, and VGG–16-based deep learning architectures have been utilized to classify the unique painting styles of various artists [116]. Similarly, an improved generative adversarial network (GAN) model has been used to capture the style of oil paintings, and compared to other methods, the suggested model provided better edge and texture features for the oil painting style migration process [117]. Such technology helps in preserving the emotional and stylistic expressions of the artwork by accurately identifying and maintaining these features. Moreover, visualization tools can track the convergence of style and content loss during training, ensuring a thorough analysis and preservation strategy. Therefore, the style features process enriched features, and any forgery or damage can potentially be identified by the intelligent vision models, if they are pre-trained on the original artworks. This method allows for a more objective evaluation of oil paintings, combining both subjective human assessments and quantitative indicators.

#### 4.2.7. Historical Features

The historical features in oil paintings preserve and convey the narratives, values, and aesthetics of the past, providing valuable insights into the events, figures, and cultural contexts that shaped history. Moreover, the historical features also depend on the evolution of the materials and media used to make the oil painting [3]. Thus, the oil painting materials significantly influence the appearance, longevity, and preservation of these artworks, which can be referred to as chemical features. Nasa et al. [118] found that the gliding of paint is caused by the incompatibility between different materials applied by the artist during micro-destructive examinations. In contrast to the simplistic model assumption that there is just one layer of pure color, historical paintings typically exhibit a more intricate combination of pigments or even possess a structure with multiple layers [119]. Therefore, for the robust extraction of historical features, it is necessary to combine both historical data and modern replicas whenever possible to compensate for the changes that happened over time to the oil paintings. This will allow for reliable preservation through the use of intelligent vision. Table 4 summarizes the contributions of various studies that have implemented these advanced methods, providing a comprehensive overview of their approaches and outcomes.

### 4.3. Case Studies Demonstrating the Effectiveness of AI in Oil Painting Preservation

**Figure 16:**
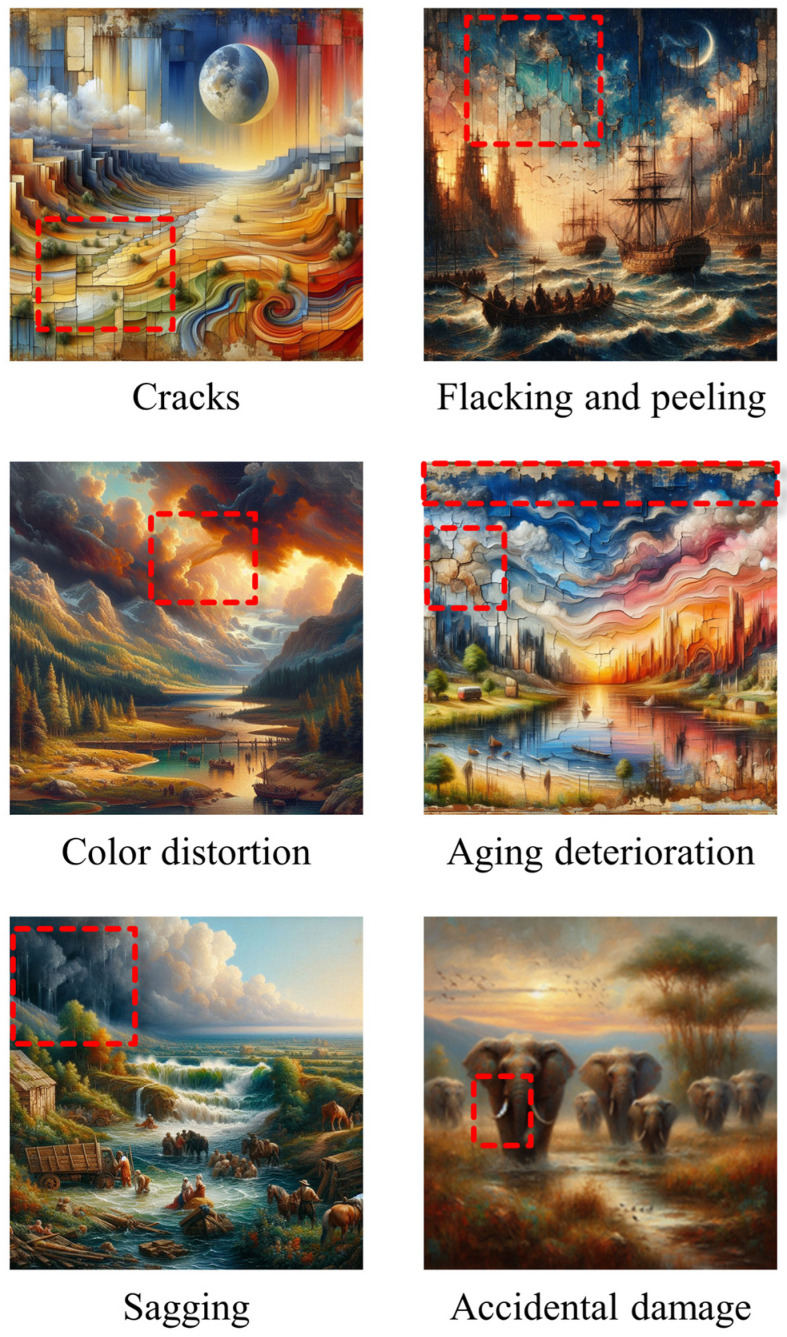
Some common damage types present in oil paintings. The red box highlights specific types of damage, providing a detailed view of their locations and characteristics.

The AI-based condition monitoring of oil painting involves feature extraction and then processing of those features through AI models for damage detection. Various features of high importance in oil paintings have been discussed in the last section. This section focuses on case studies that used the AI models to either extract the features or process the features extracted via conventional approaches. The main aim for the utilization of AI-based approaches is ultimately to identify various potential damages that can occur in oil paintings over the period or to identify forgery. Figure 16 shows some of the common damage types that can occur in oil paintings. The AI models can certainly provide evidence of any sort of damage induced in the artworks that require conservators to preserve the paintings.

**Figure 17:**
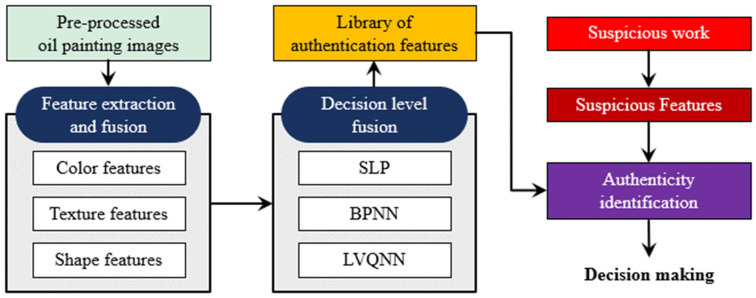
The proof of fusing color, texture, and shape features of oil paintings for authenticity identification [101].

Advances in image perception and multimedia technologies have facilitated the development of the automated analysis of oil paintings. The trend in the multimedia enterprise is the use of intelligent techniques that rely on visual perception, processing, and the identification of damage and forgery for the preservation of oil paintings. Table 5 summarizes the contribution and limitations of numerous studies that utilized AI-assisted preservation techniques for oil paintings. Wang et al. [120] employed a cross-contrast neural network model for the automated identification of oil paintings in the field of art. To accomplish this goal, a CNN model was employed to extract the distinctive characteristics of oil paintings. Subsequently, the cross-contrast probability map was computed using the contrast data to quantify the resemblance between the input images. The usefulness of the technique was demonstrated using the Selected-Wiki paintings dataset [94], which consists of over 5000 images created by 20 painters in different styles. An average accuracy of 85.75% was attained in the classification challenge involving 20 artists, surpassing the accuracy of the traditional method used for artist identification. Kuang et al. [95] utilized an advanced intelligent vision algorithm to enhance the recognition capability of oil paintings with progressively larger sizes. The proposed approach combines the identification of color and literary and scientific characteristics in oil paintings. The study determined a specific threshold value that distinguishes oil painting color and shape aspects, enabling precise identification of oil paintings. Yao and White [101] employed three neural networks, specifically the single-layer perceptron (SLP), backpropagation neural network (BPNN), and learning vector quantization neural network (LVQNN), to authenticate oil paintings. The suggested method utilizes multi-feature fusion by analyzing the creative style and extracting features related to the shape, color, and texture of oil paintings, as depicted in Figure 17. The results demonstrated that the recognition accuracy of 73% surpasses that of the existing neural networks.

**Figure 18:**
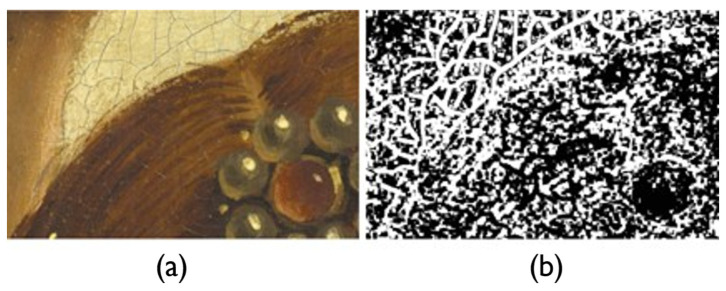
An illustrative example demonstrating the process of constructing a fusion map. (**a**) The image is the original version of a specific section from the panel called Singing Angels. (**b**) Map of fusion. Dark cracks are represented in white on the fusion map, while light cracks are represented in black (taken with permission from Ref. [121]).

The presence of cracks on painted surfaces poses a significant risk of deterioration, which, to prevent further and more severe signs of aging, must be addressed promptly. The ability to automatically detect fractures in paintings would be highly valuable for art conservators. However, traditional image processing methods are not effective in separating cracks from other lines or surface characteristics. Sizyakin et al. [121] offer a CNN-based crack detection system that can integrate many imaging modalities, including conventional imagery, infrared photography, and X-ray scans of paintings. The suggested model offers an effective way to enhance the localization of the real fracture borders using CNN, as depicted in Figure 18. A study conducted on the multimodal acquisition of the Ghent Altarpiece has revealed advances in crack detection technologies beyond the existing traditional methods in aiding art conservators. Dulecha et al. [122] proposed a pipeline for detecting cracks on egg-tempera paintings using multi-light image acquisitions. The approach is based on both single and multi-light edge detection using a CNN model that can accurately categorize image patches surrounding edge points as either crack or non-crack. The suggested approach demonstrates high accuracy in classifying crack regions, even when using single images and varying lighting orientations. The physical degradation of the artwork surface was detected by Angheluta and Chirosca [123] using deep learning models. To achieve this objective, high-resolution images were obtained by utilizing optical macro magnification. These images were then transformed into 3D reconstructions to enhance the level of detail in surface features. The objective of the research was to establish a foundation to create a cost-effective, real-time system to evaluate surface damage on artworks.

### 4.4. AI Algorithms Used for Preservation of Oil Paintings

From Table 5, it is evident that the commonly used AI models for oil painting preservation are CNN and GAN, both of which are deep learning-based. Deep learning, a subset of AI, utilizes neural networks with numerous layers to extract complex patterns and representations in data [124]. It excels in tasks such as image processing and speech recognition through vast amounts of data. In oil painting preservation, deep learning leverages its image processing capabilities for AI-assisted preservation. CNN and GAN are the two widely used deep learning models for image processing. The detailed process for implementing AI-based techniques is described in the literature [125]. Below is a brief description of both models and their implementation in oil painting preservation.

In the preservation of oil paintings, CNNs play a crucial role in analyzing and restoring digital images of artworks. These deep learning models work by scanning the painting image through multiple layers of convolutional filters, identifying features such as brushstrokes, texture patterns, and color gradients. This feature extraction helps detect areas of deterioration, like cracks or faded colors, that need restoration. CNNs can also classify and match styles across different sections of a painting, ensuring consistent restoration that respects the original artistic intent. By automating the detection and analysis process [126], CNNs aid conservators in planning precise interventions, thereby preserving the integrity and authenticity of the oil-painted artworks. Advanced CNN-based transfer learning models such as ResNet, VGG-16, VGG-19, and DenseNet-121 provide deeper architectures that help in efficient feature extraction for preserving artworks, even with limited amounts of data [127].

GANs are employed in oil painting preservation for forgery detection and style transfer of the artworks. GANs consist of two neural networks: a generator that creates new image data and a discriminator that evaluates its authenticity against real data [128]. In the context of oil painting restoration, the generator can produce realistic textures, colors, and other features of the paintings. The discriminator helps refine these features, ensuring they blend efficiently with the existing parts of the painting for style transfer or compare these features with the real paintings to authenticate the artwork or identify forgery. This process enables the creation of high-quality restorations that maintain the artwork’s original appearance and value. Using GANs, conservators can achieve detailed and accurate restorations, even with complex oil painting artworks. The performance of GANs can also be improved by using advanced architectures such as BicycleGAN, SceneryGAN, and Conditional-GAN, designed for specific applications [129]. 

## 5. Future Directions and Challenges

To advance oil painting preservation, future research should prioritize developing sophisticated AI models tailored to conservation needs. Integrating these with multispectral imaging technologies will enhance insights into materials and techniques, aiding accurate restoration. Building strong interdisciplinary collaborations between computer scientists, art historians, conservators, and material scientists is crucial for creating comprehensive models that address both artistic and material aspects of artworks. Establishing standardized, open-access databases of digital images and conservation records will support AI training and reproducibility. Additionally, ethical guidelines must be developed to ensure AI respects the authenticity and integrity of artworks. Implementing AI-powered long-term monitoring systems will facilitate early detection of changes, enabling proactive conservation. This roadmap will guide future studies, ensuring technological advancements enhance traditional conservation methods. Moreover, it can be implemented using innovative materials and methodologies, which are discussed in the following sections.

### 5.1. Utilization of Gels in Oil Painting Conservation

Gels have become essential in oil painting conservation for their precision and control in cleaning. Polysaccharide gels, such as those based on agarose and gellan gum, are particularly popular due to their biocompatibility and effectiveness in removing dirt and varnish layers from oil paintings [130]. These gels are easy to apply and can be removed without leaving residues, ensuring the integrity of the artwork is maintained. Further research into these sustainable gels is essential as their application continues to advance the field of conservation, ensuring the long-term preservation of oil paintings through safe and effective methods. Furthermore, research into nanostructured gels has opened new possibilities in the artwork conservation field. These gels, which contain nanoparticles, offer enhanced cleaning capabilities and can be engineered to respond to specific environmental conditions [131,132]. The integration of nanotechnology in gel formulations has the potential to revolutionize the conservation of oil paintings, providing conservators with powerful tools to address complex conservation challenges.

### 5.2. Exploration of Emerging Trends for Oil Painting Preservation

The conservation of oil paintings is experiencing a significant period of change due to the emergence of AI and big data technology. AI models are becoming essential in identifying microscopic cracks and invisible damages that often escape the human eye [133,134]. These advanced systems can analyze patterns and anomalies with remarkable precision, ensuring early detection and preservation. Similarly, the feature extraction and recognition of paintings is well adopted in numerous other paintings and drawings using deep learning [135]; therefore, these models need to be adopted for oil paintings as well. Moreover, the combination of conventional conservation techniques with data-driven artificial intelligence models is establishing a novel benchmark in identifying strategies to reduce, or maybe eliminate, the damage caused in different circumstances [136]. These AI techniques can serve as a tool for conservators, enhancing their traditional expertise with advanced analytical capabilities. For instance, AI-driven image analysis can be used alongside manual inspections to pinpoint damaged areas with greater accuracy. This allows conservators to focus their efforts more precisely and make informed decisions based on comprehensive data. Furthermore, integrating AI with traditional conservation methods can involve using AI models to simulate various restoration techniques, predicting outcomes and guiding the selection of the most appropriate methods. This hybrid approach ensures that the cultural and historical value of the artworks is preserved while utilizing the precision and efficiency of modern technology. Such hybridization would enhance performance, integrating the theoretical domain knowledge of human expertise with the efficiency of AI. These models would provide a promising future for the conservation of oil paintings, where technology and tradition blend to protect our cultural heritage.

### 5.3. Consideration of Ethical and Practical Challenges in AI Adoption

The integration of AI in oil painting preservation poses unique ethical and practical challenges. Ethically, it is crucial to ensure AI models are trained on diverse data sets to avoid biases that could misinterpret the artworks. In practice, the conservation community must deal with the reliability of AI assessments and the cost of implementing such technology. However, balancing the preservation of artistic integrity with the innovative capabilities of AI requires careful consideration. Incorporating AI into the preservation of oil paintings introduces the ethical dilemma of generative models being used to create fake artworks using generative adversarial networks [137]. These sophisticated algorithms can replicate the style and technique of historic painters, raising concerns about authenticity and provenance. While they offer a novel way to restore damaged pieces or recreate lost works, the potential misuse for forgery is significant. Ethical guidelines and strict regulatory frameworks are essential to govern the use of generative AI, ensuring it serves to uphold artistic heritage rather than undermine it. Thus, a collaborative approach is required that respects the historical context and material composition of artworks while harnessing the potential of AI to predict and prevent damage, ensuring the longevity of oil paintings.

### 5.4. Recommendations for Conservators, Researchers, and Policymakers Interested in Implementing AI-Driven Maintenance Strategies

For individuals involved in the fields of conservation, research, and policymaking who are interested in applying AI-based maintenance techniques for the preservation of oil paintings, numerous guidelines can guarantee successful implementation. Firstly, invest in collaborative efforts between conservation and AI experts to develop algorithms for the specific needs and challenges in preserving oil paintings. Secondly, prioritize the collection and digitization of high-quality data sets to train AI models effectively. This should be done in the modern illustration arts to further expand the potential of oil paintings [138]. Finally, establish transparent guidelines and ethical considerations for the use of AI in conservation practices. 

### 5.5. Potential Technical and Ethical Challenges

While AI technologies can provide significant benefits in oil painting preservation, they also present limitations and challenges. One major concern is the risk of AI systems misjudging the authenticity of artworks due to biases in training data or limited model interpretability, potentially leading to inappropriate restoration decisions or false attributions. To mitigate these risks, future work should focus on improving model transparency and interpretability, involving expert conservators and art historians in validation processes to ensure AI outputs align with historical and artistic contexts. Additionally, addressing ethical concerns, such as maintaining the integrity of original artworks, is crucial. Collaborative efforts between technologists and art professionals are essential to navigate these challenges and ensure AI remains a reliable and ethical tool in art conservation.

## 6. Conclusions

This review highlights the advances in the field of preservation of oil paintings from traditional to modern AI approaches. Oil paintings that offer glimpses into history, culture, and artistic excellence are susceptible to damage over time. Traditional methods, like surface treatment, structural stabilization, and surface cleaning, were reviewed for their foundational role in maintaining artwork integrity. However, the arrival of the digital information age has realized the transformation of various industries, which has also promoted developments in the field of art and painting. Therefore, this article provides an in-depth, comprehensive analysis of both traditional and AI-driven approaches to the maintenance and preservation of oil paintings. In terms of traditional preservation techniques, the article underscores varnishing, canvassing, and gel-based cleaning as pivotal methods used in the conservation of artworks. In terms of AI-driven methods, the article highlights the intelligent vision approaches that utilize image processing models, such as convolutional neural networks (CNNs) and generative adversarial networks (GANs), for the maintenance of oil paintings. Moreover, the article also provides details about the specific imagery features required by the AI models. These include color, shape, texture, edge, fractal, style, and historical features that are mainly used to predict deterioration patterns, authenticate artworks, enhance images for failure recognition, classify artistic styles, and extract historical features from oil paintings. Finally, the article discusses the future direction and challenges associated with the adoption of AI in improving the quality and sustainability of the preservation methods of oil paintings.

## Figures and Tables

**Table 1:** Overview of studies investigating mechanical damage in oil paintings.

Study	Factors	Methodology	Key Findings	Implications for Conservation
Bosco et al. [32]	RH	Analytical modeling and experimental analysis	Identified crack channeling mechanisms in historical paintings due to fluctuating humidity.	Guides conservation strategies to mitigate moisture-induced damage.
Janasa et al. [33]	Temperature and RH	Experimental testing and characterization	Studied mechanical properties and aging effects of oil paints under different conditions.	Informs strategies to stabilize painting materials against environmental fluctuations.
Jonah et al. [34]	Temperature and RH	Mechanical testing and analysis	Investigated flexibility and durability of modern painting materials under varied conditions.	Provides insights into material selection and handling practices for conservators.
Zhang et al. [35]	RH	Numerical modeling and data analysis	Developed predictive models for crack formation in paintings exposed to cyclic RH variations.	Aids in developing climate control strategies to minimize cracking in artworks.
Richard et al. [36]	Temperature and RH	Experimental monitoring during transport	Studied the effect of Silica gel on panel paintings in microclimate packages.	Provides insights to minimize damage during transport.
Carlo et al. [41]	Vibrations	IoT-based monitoring system deployment	Developed a system to measure vibrations in artworks, focusing on the prevention of mechanical damage.	Enables real-time monitoring and intervention to protect artworks during transportation and display.
Yulong et al. [42]	Vibration	Experimental modal analysis	Studied modal properties of canvas paintings to mitigate damage risks during transportation.	Provides data for safer handling and transportation protocols for delicate artworks.

**Table 2:** Overview of studies investigating chemical deterioration in oil paintings.

Study	Factors	Methodology	Key Findings	Implications for Conservation
Anna et al. [43]	Air pollution	Analysis of soiling content	Elevated sulfate levels in paintings indicative of air pollution impacts; calcium levels vary with storage conditions	Emphasizes monitoring and mitigation of indoor air pollutants to preserve artwork integrity
Maria et al. [47]	Microbial activity	Culture-dependent and -independent methods	Identification of microbial strains impacting oil paint deterioration; enzymatic activity influences material composition	Highlights the role of microbes in biodeterioration, and suggests strategies for microbial control in conservation
Santiago et al. [44]	Light exposure	Spectral aging test	Quantifies photochemical damage to paints under different lighting conditions; model correlates exposure with damage	Informs lighting standards and protocols in museums to minimize light-induced degradation
Dang et al. [53]	Pigment sensitivity to light	Experimental exposure	Evaluation of pigment color changes under museum lighting sources; identifies least-damaging light source	Provides guidelines for selecting museum lighting to preserve color integrity of paintings
Ilaria et al. [4]	Light exposure	Physicochemical analyses	Modern oil paintings exhibit fragile surfaces and heightened sensitivity to environmental factors	Effective use of gel-based cleaning methods is crucial for safely removing surface grime

**Table 3:** Traditional methods for oil painting preservation.

Study	Preservation Method	Key Findings	Limitations and Challenges
Lena et al. [64]	Novel cleaning systems	Evaluation of soft particle blasting, CO_2_-snow blasting, and Nanorestore Gel^®^ to remove embedded soiling. Nanorestore Gel^®^ shows promise in reducing pigment loss.	Limitations in scalability and accessibility; potential abrasiveness or alteration of paint surface texture.
Carretti et al. [68]	Gel-based cleaning	Developing a new class of organogel PEICO_2_ to combine the benefits of both liquid and traditional gel based cleaning of paintings.	The potential variability in performance depending on the specific composition and condition of the surface.
Porpora et al. [70]	Gel-based cleaning	Synthesizing PDMS organogel possessing high porosity and large pore sizes for controlled cleaning of artworks.	The dependency on the porosity and pore size of PDMS, which can vary significantly based on the templating agent used during its synthesis.
Usama et al. [57]	Varnishing with ZnO nanoparticles	Enhanced UV protection and microbial resistance. Reduction in color changes and improved durability against UV aging.	Challenges in uniform application and long-term stability of nanoparticle coatings.
Poli et al. [58]	Metal soaps formation	Formation of metal soaps alters paint appearance and complicates cleaning processes. Terpenic acids in varnishes contribute to carboxylate formation.	Difficulty in predicting and controlling metal soap formation over time; complicates restoration efforts.
Cecilia et al. [60]	Wax-resin impregnation and lining	Slows moisture uptake, minimizing tension-induced damage. Canvas weave and thread density critical in determining susceptibility to climate-induced shrinkage.	Challenges in achieving uniform impregnation; potential alteration of surface gloss and texture.
Poulis et al. [61]	Thermal and mechanical properties of adhesives	Comparative analysis of adhesive types (e.g., animal glue, synthetic polymers) for canvas lining. Importance of adhesive selection in maintaining structural integrity.	Adhesive aging and compatibility with historical materials; varying performance under different environmental conditions.
Kenza et al. [71]	Cleaning with triammonium citrate	Effective removal of calcium oxalate and varnish components confirmed via mid-FTIR spectroscopy.	Challenges in assessing cleaning agent residues and potential impact on long-term surface stability.

**Table 4:** The contribution of the various studies that implemented the AI-based preservation techniques for oil paintings.

Feature	Description	Technique	Application in Preservation	Refs.
Color	Conveys artist’s expression, mood, and narrative	RGB to HSV conversion	Intelligent color composition analysis	[95,96,97,98,99]
Shape	Describes geometric properties of objects	Form classification, contour retrieval	Object recognition and damage identification	[95,100,101,102,103]
Texture	Addresses visual patterns and surface properties	Statistical measures, filter-based approaches	Image segmentation and material identification	[104,105,106,107]
Edge	Provides detailed boundary information	Edge detection, Laplace operator	Crack detection and restoration guidance	[91,101,103,108]
Fractal	Analyzes intricate patterns in irregular shapes	Fractal dimension calculation	Brushstroke analysis and style classification	[105,109,110]
Style	Focuses on emotional expression or artistic styles	Neural network analysis	Artist style identification and forgery detection	[112,113,114,115,116,117]
Historic	Preserves narratives and cultural contexts	Historical data analysis	Preservation of cultural heritage and material evolution study	[3,118,119]

**Table 5:** The contribution of the various studies that implemented AI-based preservation techniques for oil paintings.

AI-Model	Preservation Type	Contribution	Ref.
Neural Networks	Craquelure (edge cracks) patterns	Analyzed a specific set of selected craquelure patterns in historical panel paintings	[21]
CNN	Restoring damaged areas	Overcoming sensitivity to black and white color causing color coverage issues in traditional methods	[26]
BicycleGAN and SceneryGAN	Improve image style transfer	Overcome limitations of existing GANs (AnimeGAN and CartoonGAN) that suffer from serious detail loss and color distortion in image migration	[27]
Conditional Generative Adversarial Networks (CGANs)	Artificial forgery detection	An automated system using CGANs and dissimilarity measurement for oil painting authentication by analyzing brushstroke styles	[85]
CNN	Illumination correction	Deep learning architecture to correct illumination in color images of paintings	[90]
SRCNN and UNet	Edge distribution detection	Style transfer algorithm for oil paintings using an SRCNN model with a UNet-based architecture, enhancing edge distribution and style emulation while overcoming blurred contours	[91]
CNN, SVM, and KNN	Improved paintings feature extraction	Overcoming the limitations in painting classification due to differences in techniques and preservation, as well as the complexity of feature extraction through improved classification of digital painting images by extracting stroke and color features	[92]
SLP, BPNN, and LVQNN	Feature extraction and artistic style analysis	Constructs a method for authenticating oil paintings using multi-feature fusion analyzing shape, color, and texture	[101]
ANN, Illustration2vec and VGG19	Emotional expression detection	An emotional expression analysis model for oil painting image optimization	[112]
VGG−16, ResNet–50, and DenseNet–121	Oil paintings classification	Introduces a novel approach using Reflectance Transformation Imaging (RTI) images to increase the accuracy of machine learning models for painting classification by incorporating visualized depth information of brushstrokes	[116]
GAN with Wasserstein distance (WGAN), and gradient penalty (WGAN−GP)	Oil painting style migration	Improved GAN models for oil painting image style migration and reconstruction, addressing issues of gradient disappearance	[117]
Cross-contrast CNN (CC-CNN)	Art identification	Feature extraction and cross-contrast probability map calculation for automated art identification in oil painting by combining image perception, processing, and identification	[120]
CNN	Crack detection	A fast crack detection algorithm based on CNN is able to integrate multiple imaging modalities and efficiently process high-resolution scans of paintings.	[121]
CNN	Crack detection	A framework for crack detection on egg-tempera paintings based on RTI data obtained with Multi-Light Image Collections (MLIC)	[122]
CNN	Cracks, blisters, and detachments detection	Accurate identification of various physical defects on polychrome artwork surfaces	[123]

## Data Availability

Not applicable.
